# A Comparison of Fitness Characters of Two Host Plant-Based Congeneric Species of the Banana Aphid, *Pentalonia Nigronervosa* and *P. Caladii*

**DOI:** 10.1673/031.010.14001

**Published:** 2010-08-23

**Authors:** Parna Bhadra, B. K. Agarwala

**Affiliations:** Ecology and Biodiversity Laboratories, Department of Zoology, Tripura University, Suryamaninagar 799130, Tripura, India

**Keywords:** host plant-specific forms, genotypes, host transfer

## Abstract

Aphids and other phytophagous insects often show intra-specific variations in relation to host plant utilization. In several instances, intra-species variations lead to host-plant specialization. These are considered to be important source of speciation. In a recent study (Foottit RG et al. 2010. *Zootaxa* 2358: 25–38) two forms of the banana aphid, *Pentalonia nigronervosa* f *typica* Coquerel (Hemiptera: Aphididae) from banana hosts and *P. nigronervosa* f. *caladii* van der Goot collected from Zingiberaceae and Araceae respectively were described as separate species, *P. nigronervosa* Coquerel and *P. caladii* van der Goot, based on morphological and molecular differences. A study was undertaken to examine the ecological and biological characters in asexual wingless morphs of the two forms of *P. nigronervosa sensu lat*. using taro (Araceae) and banana (Musaceae) as host plants. The results showed consistent differences between the two forms. In biological characters, the apterous morphs off. *caladii* from taro host plants were found to be significantly more fecund, showed a higher net reproductive rate, longer reproductive duration, and their adults lived longer than the f. *typica* aphids from banana host plants. In ecological characters, f. *caladii* aphids formed bigger colonies and in significantly less time on taro plants in comparison to f. *typica* aphids which formed smaller colonies in significantly more time on banana plants. Reciprocal transfer of the two forms of *P. nigronervosa* aphids between their host plant species lowered performance on the transferred host plants. These results confirmed that *P. nigronervosa* f. *typica* from banana hosts and *P. nigronervosa* f. *caladii* from taro hosts are indeed two different species in relation to host plant utilization and suggested that the observed differences in their fitness characters represented distinct genotypes.

## Introduction

The majority of the phytophagous insects live in heterogeneous environments that consist of varied host plants, climate and biological conditions. As a result, these insects often show variations in morphological, biological and ecological attributes of their populations (Singh and Cunningham 1981; Helden et al. 1984; Powell et al. 2006). Often this can cause problems in the accurate separation of taxa at species and infra-species categories (Hille Ris Lambers 1966; Miyazaki 1987; Raychaudhuri 1980; Agarwala and Ghosh 1985). Six distinct host-related populations of *Aulacorthum solani* (Damsteegt and Voegtlin 1990), three populations of *Lipaphis pseudobrassicae* (Agarwala et al. 2009) and four distinct hostspecialized populations of *Aphis gossypii* (Agarwala and Das 2007) were identified based on variations in morphology and ecological performance. Gorur et al. (2005) recorded genotypic variability and phenotypic plasticity in *Aphis fabae* when reared on preferred host as well as on novel host. Genotypic variability and phenotypic plasticity in host plant choice behavior of *A. fabae* were also recorded when tested on field host plant and novel host plant (Gorur et al. 2007).

In this study the fitness of populations of the banana aphid, *Pentalonia nigronervosa* Coquerel (Hemiptera: Aphididae), collected from two different host plant species, the banana, *Musa paradisiaca* L. var. champa (Zingiberales: Musaceae) and taro, *Colocasia esculenta antiquorum* L. (Arales: Araceae) were examined in terms of their ecological and biological characters. Worldwide, *P. nigronervosa* is recognized due to its vector populations on banana plants (Rajan 1981; Hu et al. 1996; Thinbhuvanmala et al. 2005; Robson et al. 2007). This aphid species is known to occur in two morphs from this part of the world, an apterous parthenogenetic viviparous female morph, and an alate parthenogenetic viviparous female morph (Raychaudhuri 1980; Blackman and Eastop 1984; Lorterio 1993). Banana and taro plants occur in the wild as well as in cultivation in large parts of east and north-east India and, therefore, are interspersed and offer *P. nigronervosa* populations opportunities to adapt to these hosts. Eastop (1966) reported two forms of *P. nigronervosa* on the basis of morphological differences in the alate viviparous morph from Australia and other parts of southern hemisphere, (i) *P. nigronervosa* f. *caladii* van der Goot from taro and (ii) *P. nigronervosa* f. *typica* from banana. Siddappaji and Reddy (1972) reported that the aphids from banana plants in parts of southern India belonged to the form *typica* Eastop, and those infesting cardamom and taro plants belonged to the form *caladii*. These host plant species provided different food environments for colonisation by aphids. Banana plants are infested by aphids at the bases of the uppermost cigar-shaped leaves and in the spathe, whereas taro plants are infested on the stem near the root and seldom at the bases of broad open leaves. In the environment of north-east India, distinctive features of the two host plant species provided ideal conditions for sympatric populations of *P. nigronervosa*.

The occurrence of two distinct forms of *P. nigronervosa* in the same habitat suggests that these insects might differ in their developmental and reproductive fitness in different host environments. No information was available about the evaluation of fitness characters in terms of biological and ecological performance of these aphids on taro and banana host plants (Eastop 1966; Rao and Naidu 1973; Foottit et al. 2010). In a recent study the two forms of the banana aphid, f. *typica* from banana hosts and f. *caladii* from hosts of Zingiberaceae and Araceae were described as separate species, *P. nigronervosa* and *P. caladii*, respectively, based on large and consistent differences in morphometry and molecular analysis (Foottit et al. 2010). Assuming the results of Foottit et al. (2010) would also be true for biological and ecological characters of the two species, a study was undertaken in laboratory reared clones of *Pentalonia* aphids from *M. paradisiaca* var. champa (banana host) and *C. esculenta antiquorum* (taro host). Biological characters including development and reproduction, and ecological characters including maximum population size, and growth rate of the clonal populations of *P. nigronervosa* from banana and taro hosts were investigated. Colonization success of aphids of respective clones from their field hosts to laboratory hosts were also tested in reciprocal host transfer experiment. The objective of this study was to record population and developmental parameters of the two species of *Pentalonia* described by Foottit et al. (2010) that could have bearings on the fitness of these aphids on their respective host plants and provide data that could test their identity as distinct species.

## Materials and Methods

### Insects

Apterous parthenogenetic viviparous aphids of *P. nigronervosa* and *P. caladii* were collected from banana and taro plants, respectively, found in the wild at five different locations, separated by about 2000 m distance from each other, in and around Agartala, north-east India (23.50°N latitude and 91.25°E longitude). These aphids were used to raise ten stock cultures, five each of *P. nigronervosa* on banana and *P. caladii* on taro host plants, under greenhouse conditions (24 ± 1° C temperature and 16:8 L:D photoperiod).

Host plants of the two species in early vegetative stage were maintained individually in clay or plastic pots and these were held in water trays on benches illuminated with photo-synthetically active radiation lamps. Individual plants, two from each location, were infected with a single fourth instar apterus aphid collected from their respective locations in the fields. These were allowed to grow, reproduce and increase in number. Aphid cultures on individual potted plants were confined in nylon net cages in segregated locations. This was repeated ten times for each plant species. All aphids produced from a single mother on each of the plants by this practice consisted of same genotype and, thus, constituted a clone. Fourth instar aphids produced of the same genotype of a grandmother on a plant species were used in experiments. Individual aphids, chosen randomly from banana and taro clones in the greenhouse, were placed on the apical part of the 16–20 day old pot-grown saplings at the early vegetative stage in a rearing cabinet (temperature: 24 ± 1° C; 65% RH and 16:8 L:D photoperiod). Thus, several sister clones of the same genetic lineage of the two aphid species were raised on their two host plant species. Aphid-infected individual plants were individually caged to avoid any contamination during the experiment. Observations were made at frequent intervals until each clone attained its maximum increase in population and then started to decline. Sister clones were monitored individually several times in a day. Alate females were discarded. Aphids from these clones representing two different genotypes from the two host plant species were used to measure differences in their ecological and biological characters. For determining the mean relative growth rate, parthenogenetic females of both the aphid species from their clones were placed individually in leaf cages (Blackman 1987) to obtain parthenogenetic descendents. Individual aphids were monitored for weight at birth (<12h) and at the final molt during their development.

### Population parameters

Maximum population size and growth rate for the two aphid species were determined from their respective host plant species. Twenty replicates were used in each study.

Maximum population size (*N_t_*) of a clone achieved on a potted plant and the time taken to reach the *N_t_* (*T*) were used to compare any difference in the performance of *P. nigronervosa* and *P. caladii* on their respective host plants.

Population growth rate (GR), the increase in number of aphids of a clone per day per plant in the rising phase of population increase, was calculated by the formula
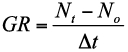

where *N_t_* is the number of aphids present at the maximum count of the population on a plant, *N_o_* is the number of aphids initially released on a potted plant, and Δ*t* is the difference of time between *N_0_* and *N_t_* (Odum 1971).

#### Developmental parameters

Biological characters including developmental time (DT), generation time (GT), reproductive duration (RD), fecundity (F) and adult longevity (AL) were recorded for individual aphids of the two *P. nigronervosa* genotypes.

For this purpose individual third or fourth stadium nymphs were each placed on a detached leaf in a leaf cage (Blackman 1987) in a temperature controlled cabinet at 24±1° C. This was repeated ten times for aphids from the two host species. Nymphs were allowed to become apterous adults, to reproduce in the first 24 hours and then the adults were removed. Only one new born aphid of an adult was retained and the rest removed. Its weight was recorded and allowed to develop to the final molt when it was weighed again and observed for the durations of prereproduction, reproduction and postreproduction. The number of nymphs born to individual aphids was counted and all but one was removed. The remaining aphid was allowed to develop into second generation. Leaves were changed every 24 h to maintain the vigor of the experimental culture. As a result of this procedure, birth weight (BW) of nymphs within 12h of laying by a mother aphid, adult weight at the final molt (AW), developmental time from the birth of a nymph to its final molt, generation time from the birth of a nymph to the time of onset of reproduction by this nymph, reproductive duration from the birth of the first nymph to the last nymph by an apterous female, fecundity and adult longevity were recorded.

The mean relative growth rate (MRGR), a measure for assessing the performance of different clones of a species under different environmental conditions (Radford 1967), was determined following the method of Watt and Hales (1996):
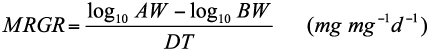

and expressed as mg increase in weight of aphids born per mg of the mother aphid per day.

Net reproductive rate (R0), the multiplication rate of an aphid in a clone per generation, was calculated using the equation (Krebs 1985),
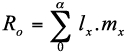

where *l_x_*is the proportion of females surviving, and *m_x_* is the number of female offspring born per female during its reproductive time.

Intrinsic rate of increase (*R_max_*), a measure of rate of increase of a population under controlled conditions, was calculated using the formula,
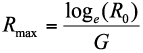

where *R_0_* is the net reproductive rate and *G* is the mean length of a generation determined by the equation (Krebs 1985),
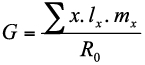



### Host transfer experiment

Aphids of the two species were subjected to reciprocal transfer of hosts to record the colonization success in a new food environment. Individual nymphs, 0–12 h old, were released at the apical most part of potted plants of similar age of field hosts (control) and laboratory hosts (treatments). These aphids were allowed to settle and produce nymphs for the second generation. If successful, a third generation was produced. Two treatments were set up simultaneously using parental clones of *P. nigronervosa* and *P. caladii* from their respective host plants. In the first treatment, *P. caladii* aphids were transferred individually from the taro field host to the laboratory host, banana, and in the second treatment *P. nigronervosa* aphids were transferred from the banana field host to taro plants as the laboratory host. In both the treatments, performances of aphids on their respective field host plants were considered as the controls. In each case of host transfer, ten replicates were used to record the success rate of survival and reproduction by apterous viviparous aphids on a host plant leading to the establishment of a colony. Aphids that either failed to develop to the adult stage in the first generation or failed to produce second or third generation progeny were considered to be unsuccessful.

### Data analysis

Data of the third generation aphids were used to compare results of population and developmental parameters. This was done to allow the aphids sufficient time for acclimatization to the laboratory rearing conditions. All weights in this study were taken using a Mettler microbalance (www.met.com) sensitive to 2µg. Each of the population and developmental parameters that were measured from the wingless aphids of the two *Pentalonia* species were compared using the Student *t* test. Origin 7 (www.originlab.com) was used for the analysis of data.

## Results

### Population parameters

**Figure 1.**
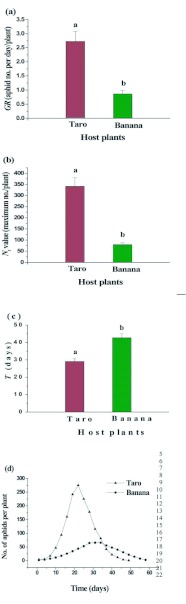
Mean values of growth rate (GR) (a), maximum population size (*Nt*) (b), time to attain *N_t_* (*T*) (*c*) and population trend (d) of *Pentalonia caladii* and *P. nigronervosa* determined on potted plants of taro and banana, respectively. High quality figures are available online.

Clones of *P. nigronervosa* and *P. caladii* showed significant differences in growth rates and maximum population size on their respective host plants. Time taken to attain the maximum population size also differed between the clones of the two aphid species from the two species of hosts. The *P. caladii* clones reared on taro plants showed a mean growth rate of 2.73 aphids/ day which was higher by approximately 3.14 times compared to the clones of *P. nigronervosa* reared on banana plants (0.87 aphids/ day; Figure 1a). The maximum population size of *P. caladii* clones on taro plants for was 341.89 aphids per plant which was 4.27 times higher than the mean maximum population size of *P. nigronervosa* clones on banana plants (80.10 aphids per plant; Figure 1b). However, the time taken by the clones of the two species of *Pentalonia* to achieve the maximum population size on their respective host plants did not show the same trend. Aphids of *P. caladii* clones on taro plants reached the asymptote in significantly less time of 29.20 days, which was lower by 68%, in comparison to the *P. nigronervosa* clones reared on banana plants (42.80 days; Figure 1c). Thus, *P. nigronervosa* clones on banana plants formed smaller colonies more slowly in comparison to *P. caladii* clones on taro plants which formed bigger colonies in less time (Figure 1d).

### Developmental parameters

Apterous aphids of *P. caladii* and *P. nigronervosa* clones from taro and banana plants, respectively, showed significant differences in development time, generation time and fecundity and these were distinguishable in terms of their minimum and maximum values from the respective means (Table 1). The *P. caladii* aphids from taro plants took longer to complete development (development time: mean ± SEM = 10.05 ± 0.20 days) and to begin reproduction (i.e., generation time: mean ± SEM = 11.35 ± 0.25 days) than by the *P. nigronervosa* aphids on banana plants (development time: mean ± SEM = 8.25 ± 0.11 days; generation time: mean ± SEM = 9.45 ± 0.17 days). Between the two aphid species, individual aphids from the *P. caladii* clones from taro host showed longer reproductive duration (mean ± SEM = 13.50 ± 0.97days) and longer adult longevity (mean ± SEM = 16.25 ± 1.15 days) in comparison to aphids from the *P. nigronervosa* clones (mean ± SEM: reproductive duration = 9.60 ± 0.37 days; adult longevity = 12.85 ± 0.40 days). Aphids from the taro clones were 2.24 times more fecund (mean ± SEM = 24.50 ± 1.06 progeny per female) and showed higher net reproductive rate (mean ± SEM = 24.56 ± 0.90) than the aphids from the banana clones that showed significantly lower fecundity (mean ± SEM = 10.90 ± 0.38 progeny per female) and lower net reproductive rate (mean ± SEM = 10.71 ± 0.66). Intrinsic rate of increase and mean relative growth rate of *P. nigronervosa* and *P. caladii* aphids on the two plant species, however, did not show comparable differences (Table 1).

### Host transfer experiment

**Figure 2.**
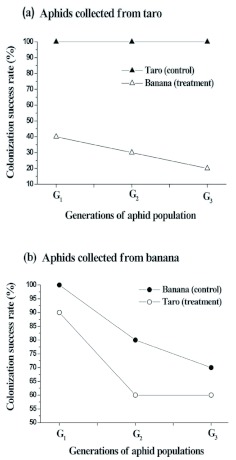
Success of colonization by *Pentalonia caladii* and *P. nigronervosa* species of aphids through generations on their field hosts (control) and across host plants, (a) treatment I: *P. caladii* transfered to laboratory host banana, (b) treatment II: *P. nigronervosa* transfered to laboratory host taro. High quality figures are available online.

When transferred to banana plants, the colonization success rate of *P. caladii* aphids from taro plants (treatment I) declined from 40% in the first generation to 20% in third generation. In each of the three generations, 60% or more of the aphids transplanted from taro plants either did not survive to produce offspring or perished on the transferred host plant (Figure 2a). When aphids of *P. nigronervosa* aphids from banana host were transferred to taro host (treatment II), the colonization success rate was found to be 90% in the first generation and declined to 60% in the second and third generations. In both cases of host transfer, performance of aphids of the two *Pentalonia* species declined on the alternate host plant species in second and third generations after initial success in the first generation (Figure 2b).

**Table 1.**
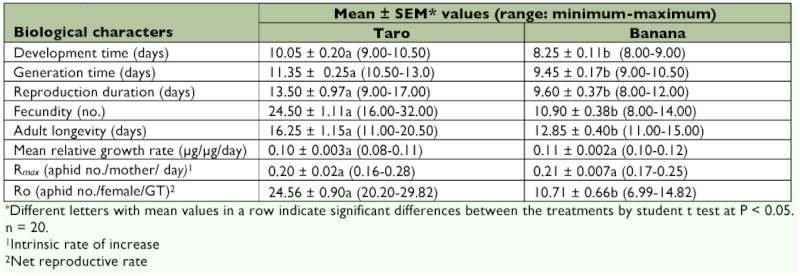
Mean values of biological characters studied in *caladii* and *typica* phenotypes of *Pentalonia nigronervosa* from taro and banana host plants.

## Discussion

This study has provided evidence that *P. nigronervosa* and *P. caladii* aphids show strong differences in their developmental and reproductive fitness in relation to banana and taro host plants, respectively, which supports the existence of two distinct lineages proposed by Foottit et al. (2010). Apterous viviparous morph of *caladii* from taro host plants were found to be significantly more fecund, showed higher net reproductive rate, longer reproductive duration, significantly longer development and generation times, and their adults lived longer than the *P. nigronervosa* aphids from banana host plants. In population parameters, *P. caladii* aphids on taro plants formed bigger colonies in significantly less time in comparison to *P. nigronervosa* aphids that formed smaller colonies more slowly on banana plants. Although the observed differences in population parameters of the two *Pentalonia* species are large on their respective host plants, these differences could be attributed, at least in part, to the differences in phenology of the two host plants which are quite different in terms of their relative sizes and growth rates. Thus, under field condition, aphids of the two congeneric species could show population growth in response to phenology of their respective host plants which might be different than that observed in the laboratory study (Agarwala and Datta, 1999). Nevertheless, large and consistent differences recorded in the performance of the two *Pentalonia* species on banana and taro hosts in the controlled environment of the laboratory are indicative of inherent differences in their development and growth ability. Biotic potential (*R_max_*) of the two species on their respective host plants, as evident from the intrinsic rate of increase was, however, found to be similar (Table 1).

Results from host plant transfer experiment on reciprocal basis suggested that performance of both the species of *Pentalonia* considerably declined in successive generations on the alternate hosts in comparison to the control hosts and the decline was found to be more profound in *P. caladii* than in *P. nigronervosa* aphids. These results suggested that host plant-aphid interaction in the populations of *Pentalonia* has lead to the evolution of more than one species based on their fitness in the distinct environment of these two host plants and these represented different species as described by Foottit et al. (2010). However, the status of *Pentalonia* populations reported from *Dieffenbachia* spp. (Family, Araceae) (Blackman and Eastop 1984) remain to be ascertained for their true species status. The only other valid species known in the genus is *P. gavarri* Eastop described from alate viviparous morph collected in yellow traps from Philippines and north east Australia (Eastop 1967; Carver and Hales 1983). Subsequently, apterous viviparae of this species was described from west Malaysia infesting graminaceous hosts (Martin, 1987).

Both species of *Pentalonia* in this study are commonly known by their alate and apterous viviparous morphs and to reproduce by asexual means in the environment of northeast India and elsewhere in their distribution range of tropical and subtropical regions (Eastop, 1966; Miyazaki, 1971; Raychaudhuri, 1980; Robson et al. 2007; Foottit et al. 2010). The rare apterous oviparous morph of *P. nigronervosa* from *Curcuma domestica* Valeton (Zingiberaceae) recorded from the tropical plains of West Bengal, and also in neighbouring Nepal (Bhanotar and Ghosh, 1969; Blackman and Eastop, 1984) seem to suggest that these aphids are endowed with the genetic potential of producing sexual forms in warm climate similar to aphids of Greenideinae, several species of which are known to produce sexual morphs in the hot summer of India and Australia (Agarwala and Dixon, 1986; Dixon, 1998). In Japan, *P. nigronervosa* is reported from the southernmost parts, Okinawa, which is warmer compared to central and northern parts which have temperate climate (Miyazaki, 1971). With the application of modern practices of horticulture and agriculture, these plants, particularly banana, are now grown throughout the year, because of their economic importance; thereby the importance of seasonality of host plants appears to have less bearing on the life of aphids except for the influence of shorter a photo period and lower temperature that are known to provide necessary stimuli for the production of sexual morphs (Dixon, 1998). In the area of this study and elsewhere in the distribution range of *Pentalonia* species, necessary climatic stimuli to produce sexuparae are absent. Therefore, it appears reasonable to assume that these aphids, like some other aphid species, have been evolving in response to the host plant environment (Blackman and Spence, 1992; Gorur et al. 2005; Fenton et al. 2009).

A number of studies of insect herbivores have found significant intra-specific variation in characters associated with host plant utilization (Futuyama and Philippi 1987; Via 1990). It has been shown that intra-specific variation or differences in congeneric species of aphids can be due to either one or the combined action of genetic differences, effects of host plant species (Lowe 1973; Via 1991; Gorur et al. 2005) and/ or facultative endosymbionts (Tsuchida et al. 2006). The two species of *Pentalonia* tested in this study, showed large and consistent differences in fitness characters on their respective host plants. Such differences in response to host plants is suggestive of strong genotype (aphids)-environment (host plants) interactions indicating increased genetic variation. The hypothesis of sympatic speciation in phytophagous insects occurring via phenotypic host race formation has been gaining acceptance in recent years (Butlin 1995; Gorur 2000; Agarwala and Das 2007; Agarwala et al. 2007, 2009). The result of this study has contributed to our understanding as to how phenotypic plasticity facilitates speciation in aphids (Agarwala, 2007; Gorur et al. 2007) through host plant specialization (Fenton et al. 2009).
